# Possible Therapeutic Application of Targeting Type II Natural Killer T Cell-Mediated Suppression of Tumor Immunity

**DOI:** 10.3389/fimmu.2018.00314

**Published:** 2018-02-22

**Authors:** Shingo Kato, Jay A. Berzofsky, Masaki Terabe

**Affiliations:** ^1^Department of Gastroenterology and Hepatology, Yokohama City University School of Medicine, Yokohama, Japan; ^2^Molecular Immunogenetics and Vaccine Research Section, Vaccine Branch, Center for Cancer Research, National Cancer Institute, National Institutes of Health, Bethesda, MD, United States

**Keywords:** natural killer T cell, type II natural killer T cell, tumor immunology, immune regulation, immunosuppression, immunotherapy, lipid antigens, transforming growth factor beta

## Abstract

Natural killer T (NKT) cells are a unique T cell subset that exhibits characteristics from both the innate immune cells and T cells. There are at least two subsets of NKT cells, type I and type II. These two subsets of NKT cells have opposite functions in antitumor immunity. Type I NKT cells usually enhance and type II NKT cells suppress antitumor immunity. In addition, these two subsets of NKT cells cross-regulate each other. In this review, we mainly focus on immunosuppressive NKT cells, type II NKT cells. After summarizing their definition, experimental tools to study them, and subsets of them, we will discuss possible therapeutic applications of type II NKT cell pathway targeted therapies.

## Introduction

One of the successful recent approaches to cancer immunotherapy is to overcome immunosuppressive signaling pathways, such as the CTLA-4 or PD-1 pathways. These pathways are endogenous regulatory systems to suppress excessive immunity. Since tumor antigens (Ags) are autologous, antitumor immunity is a kind of autoimmune reaction and is inhibited by those immunosuppressive mechanisms. Thus, to induce antitumor immunity of sufficient magnitude, it is important to overcome them.

In the tumor microenvironment, multiple immune cells form an interacting network. In addition, considering that the suppression mechanisms differ among the mouse tumor models, the mechanisms may also differ among cancer types in humans. Moreover, it is known that even cells of the same mouse tumor cell line growing in different organs are subject to different dominant immunosuppressive mechanisms (1). These findings indicate that the most appropriate immunotherapy for cancer patients with distant metastasis may be different among the metastatic sites of cancer. Altogether, a detailed understanding of suppression mechanisms is important to establish appropriate strategies to control them.

In this review, we mainly focus on immunosuppressive natural killer T (NKT) cells, type II NKT cells. Type II NKT cells play a suppressive role in many diseases, including autoimmune and inflammatory diseases, as well as the tumor setting. Although multiple experimental tools have been used, no tools can analyze the entire population of type II NKT cells so far. Thus, when we discuss type II NKT cells, it is important to understand the advantages and limitations of each experimental tool, as well as the definition of type II NKT cells used in the study.

## Definition of NKT Cells, Type I NKT Cells, and Type II NKT Cells

Natural killer T cells are a unique T cell subset that exhibits characteristics from both innate immune cells and T cells. Similar to innate immune cells, NKT cells react quickly to stimuli and produce a large amount of various kinds of cytokines and chemokines to modulate the immune response (2, 3). Also, like T cells, NKT cells express a T cell receptor (TCR) and respond in an Ag-specific manner. Thus, the nomenclature of NKT cells may be misleading, as they are not related to natural killer (NK) cells. The name originally came from the expression of the NK1.1 marker by many of them (4), but this was an unreliable characteristic and the definition was changed to define any T cell expressing a TCR that recognized a lipid presented by CD1d as an NKT cell (5).

With this feature of lipid–CD1d specificity, NKT cells can be distinguished from conventional T cells that recognize peptide Ags presented by conventional MHC molecules. Therefore, NKT cells expand the repertoire of Ags that the cellular immune system can recognize beyond the proteins detected by conventional T cells. Thus, the current definition of NKT cells is any CD1d-restricted T cell, which can recognize lipid Ag presented by CD1d through its TCR.

Type I NKT cells are defined as CD1d-restricted T cells, which express a TCR α-chain that utilizes Vα14-Jα18 gene segments in mice and Vα24-Jα18 gene segments in humans. This semiinvariant TCR α-chain was initially discovered as a quite unusual TCR α-chain across several hybridoma lines (6), because the TCRα chain expressed by these NKT cells has very few or no nucleotide insertions in the CDR3 region. Thus, type I NKT cells are also referred to as invariant or iNKT cells.

Type II NKT cells are defined as CD1d-restricted T cells that express diverse TCR α-chains other than the semiinvariant one expressed by type I NKT cells. This definition means all CD1d-restricted T cells except for type I NKT cells are type II NKT cells. Thus, type II NKT cells can be a mixture of a variety of different subsets. However, currently, no experimental tools exist to identify or analyze the entire population of type II NKT cells. When we discuss type II NKT cells, it is important to understand the advantage and limitation of each experimental tool, as well as the definition of type II NKT cells in the discussion. The frequency of the entire type II NKT cell population is not known yet. However, sulfatide-reactive type II NKT cells are reported to be approximately 4.5% of the mononuclear cell fraction of cells in the liver (7). Taking into consideration that these type II NKT cells have the same Ag specificity in naive mice, this T cell population is not trivial in size compared to the population of naive conventional T cells specific for a single Ag or epitope. In this review, we first summarize the experimental tools and subsets of type II NKT cells, and next, we will focus on their role in tumor immunity.

## Experimental Tools for Type II NKT Cell Analysis

Type II NKT cells were originally discovered from MHC-II-deficient mice. Cardell et al. discovered that MHC-II-deficient mice unexpectedly had a significant population of peripheral CD4^+^ T cells (8), even though conventional CD4^+^ T cells are absent. The authors created several CD4^+^ T cell hybridomas from MHC-II-deficient mice and found that many of them were CD1d restricted. These type II NKT cell hybridomas are useful tools for *in vitro* analysis. For example, many lipid Ags for type II NKT cells have been discovered using NKT cell hybridomas (9–11). Also, analysis using type II NKT hybridomas demonstrated that type II NKT cells did not recognize α-galactosylceramide (α-GalCer), which is a potent stimulator for type I NKT cells (12), suggesting that the lipid Ags recognized by type I NKT cells and type II NKT cells are different.

For *in vivo* analysis, the function of different NKT cell subsets has been assessed by comparison of immune responses of WT mice that have both type I and II NKT cells to those of Jα18^−/−^ mice that lack type I NKT cells but retain type II and to those of CD1d KO^−/−^ mice, which lack all NKT cells. Although this model can provide only indirect evidence of type II NKT cell function, currently, this is the only strategy that can analyze the *in vivo* function of the entire type II NKT cell population.

For direct analysis of type II NKT cells, three experimental tools have been reported, 24αβ-TCR transgenic mice, 4get Jα18^−/−^ mice, and lipid Ag-loaded CD1d tetramers. Although none of them can identify the entire population of type II NKT cells *in vivo*, these tools can provide direct evidence of *in vivo* function of at least a subset of type II NKT cells. These experimental tools are summarized in Table 1.

**Table 1:** Experimental tools to analyze type II NKT cells.

Tools	Advantage	Limitation
Type II NKT cell hybridomas	Easy to handle	Limited to *in vitro* experiments and specific clones available, not representative of all populations

Comparison of WT mice, Jα18^−/−^ mice, and CD1d KO^−/−^ mice	This model can provide *in vivo* behavior of the entire type II NKT cell population	This model can provide only indirect evidence of type II NKT cell function

24αβ-TCR transgenic mice	This model enables identification of type II NKT cells *in vivo*	This model can provide behavior of type II NKT cells with one TCR repertoire, not representative of the majority of type II NKT cells
	This model can provide *in vivo* behavior of type II NKT cells	The majority of other T cells are absent in this model

4get Jα18^−/−^ mice	This model enables identification of type II NKT cells *in vivo*	Not all type II NKT cells may be identified in this model, only ones in which the IL-4 gene is activated
		Type I NKT cells are absent in this model
	More conventional T cells are present than in 24αβ-TCR transgenic mice	Once other T cells are activated, type II NKT cells can no longer be distinguished from other T cells as other T cells may express GFP

Lipid antigen-loaded CD1d tetramers	These tools can provide direct identification and evidence of type II NKT cell function	Currently, no reagents can identify all type II NKT cells, just ones with receptors recognizing the lipid–CD1d combinations available
		Some reagents are technically difficult to create

*NKT, natural killer T cell; TCR, T cell receptor*.

## Sulfatide-Reactive Type II NKT Cells

**Figure 1:**
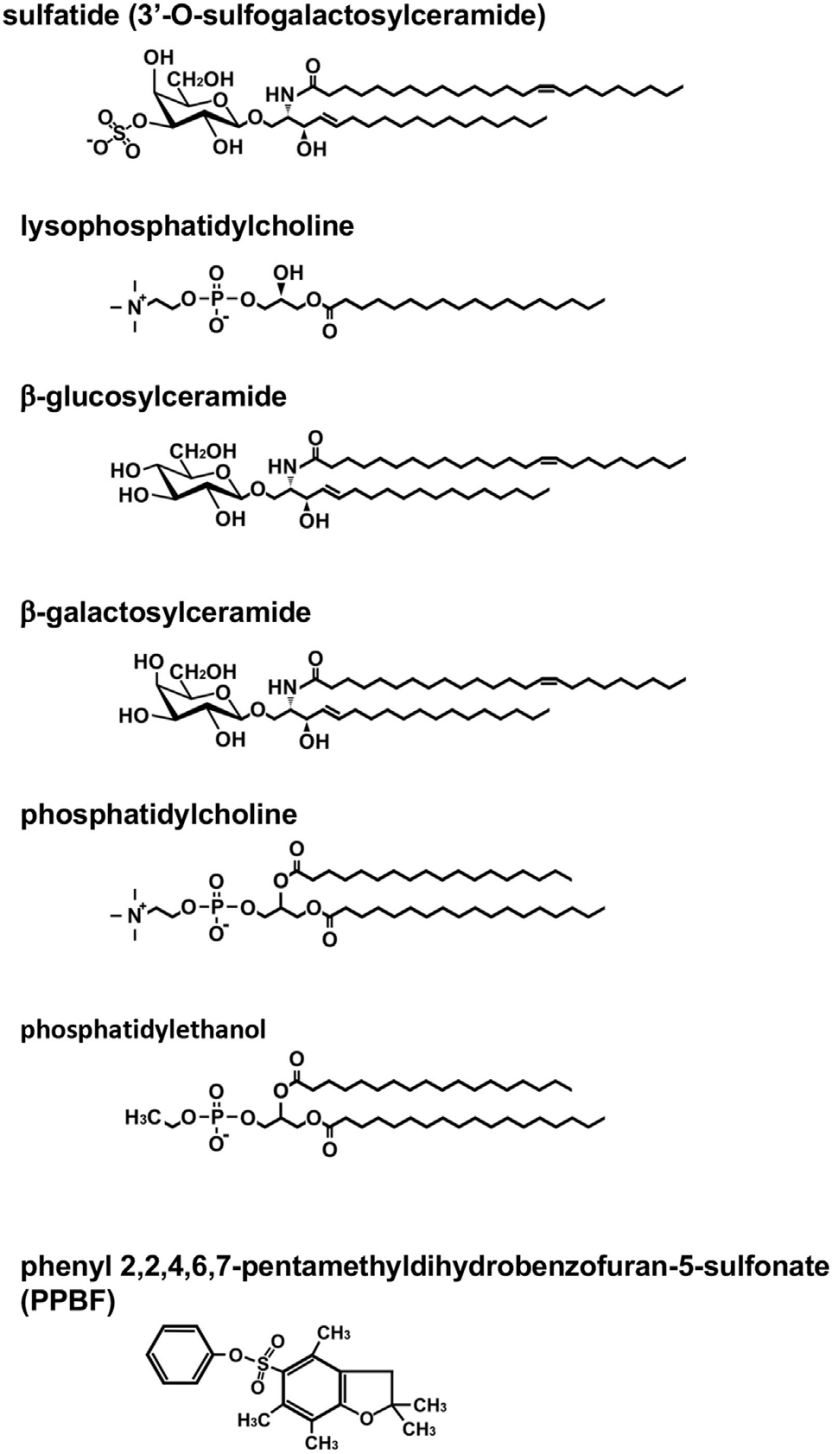
Structure of lipid antigens for type II natural killer T (NKT) cells. Type II NKT cells can recognize a broad range of both endogenous and exogenous lipid antigens. The representative structures for each lipid are shown. Pollen grain phospholipids, such as phosphatidylcholine and phosphatidylethanol, are recognized by human type II NKT cells.

The first lipid Ag for murine type II NKT cells, sulfatide, was reported in 2004 (7) (Figure 1). Sulfatide, 3′-*O*-sulfogalactosylceramide, is an endogenous glycolipid, which is abundant in the myelin in the nervous system, as well as the pancreas, kidney, and liver (13). Notably, the authors created sulfatide-loaded CD1d tetramers and identified sulfatide-reactive type II NKT cells in the liver and spleen. This is the first report that type II NKT cells in *ex vivo* mononuclear cells were visualized. Subsequently, using sulfatide-loaded CD1d tetramers, the TCR repertoire of sulfatide-reactive type II NKT cells in the liver was analyzed (14). As expected, the TCR repertoire of sulfatide-reactive type II NKT was diverse, but most frequently employed alpha gene segments from Vα1 and Vα3 and paired with Vβ8.1/Vβ8.3.

Although sulfatide-loaded CD1d tetramers were reported in 2004, the analysis of sulfatide-reactive type II NKT cells has not been as rapid as that of type I NKT cells. This may be partly due to the fact that sulfatide-loaded CD1d tetramers are not widely available, because making stable sulfatide-loaded CD1d tetramers to stain sulfatide-reactive type II NKT cells is technically difficult. Recently, we have overcome these problems (Kato et al., manuscript in preparation). We found that a significant number of sulfatide-reactive type II NKT cells exist in the lung, which is a major target organ for tumor metastasis. This population can produce IL-13 after activation, consistent with the previous observation in the analysis of their suppressive effect in tumor immunity (15). A transcriptome analysis of sulfatide-reactive type II NKT cells indicated that this cell type has a gene expression profile distinct from but similar to that of type I NKT cells, in contrast to Th2, Th0, and innate-like lymphoid cells (ILCs)/NK cells.

## 24 αβ-TCR Transgenic Mice

The 24αβ-TCR was identified as one of the TCRs in the repertoire of murine type II NKT cells from the CD4^+^ type II NKT cell hybridoma VIII24 that expresses a Vα3.2 and Vβ9 rearrangement (8). For *in vivo* analysis of type II NKT cells, TCR transgenic mice carrying the 24αβ-TCR were developed (16). In 24αβ-TCR transgenic mice, the majority of αβ-T cells express the 24αβ-TCR. They express NK1.1, CD122, intermediate levels of TCR and are CD4/CD8 double negative or CD4^+^. Upon activation *in vitro*, they secrete large amounts of IL-4 and IFN-γ, as this is characteristic of NKT cells.

The gene expression profiling of 24αβ-TCR cells revealed that the cells expressed genes predominantly associated with Th1 effector functions comparable to type I NKT cells (17). Also, the 24αβ-TCR cell signature genes, such as annexin A2, Ly6C and c-kit, have all been shown to be augmented in CD8αα thymic cells obtained by re-aggregation thymic organ culture (18), which have self-reactive TCRs and phenotypic characteristics of innate immune cells. Taken together, the pattern of genes highly expressed in 24αβ-TCR cells indicates that the cells have characteristics of innate immune cells and Th1 cells.

Although the gene profile suggests that 24αβ-TCR cells have features of cytotoxic T cells (CTLs), their immune-suppressive effect has been reported in multiple mouse disease models, including type I diabetes (19) and autoimmune colitis (20). The functions of 24αβ-TCR cells in tumor settings are unclear.

Although VIII24 was reported not to react to sulfatide, interestingly, we found that lung sulfatide-reactive type II NKT cells were partially positive for Ly6C and c-kit, similar to 24αβ-TCR cells (Kato et al., manuscript in preparation). These findings suggest that these two subsets of type II NKT cells may share some characteristics.

## 4get Jα18^−/−^ Mice

Other approaches to detect type II NKT cells *in vivo* involve using constitutive expression of cytokine mRNA for their marker. The IL-4 GFP enhanced transcript (4get) mice were used to identify type II NKT cells *in vivo* based on the hypothesis that similar to type I NKT cells, which constitutively express IL-4 mRNA, type II NKT cells must express IL-4 at a steady state (21, 22). TCRβ^+^GFP^+^α-GalCer/CD1d tetramer-negative cells were sorted from liver mononuclear cells of 4get Jα18^−/−^ mice. This population produced IFN-γ when co-cultured with CD1d-expressing bone marrow-derived dendritic cells (DCs), suggesting the cells reacted with self-Ags presented by CD1d, and thus they were type II NKT cells. This sorted population reacted with lipid ligands, which had been previously shown to be ligands for type II NKT cells, such as β-glucosylceramide (β-GlcCer) and β-GalCer (10, 23) (Figure 1). The sorted β-GlcCer reactive type II NKT cells did not respond to sulfatide and favored TCR gene segments from Vα8 and Vβ8.1/8.2, a combination that is distinct from sulfatide-reactive type II NKT cells (22).

## Other Type II NKT Cells

Type II NKT cells recognize both glycolipids and phospholipids derived from self as well as microbes. In addition to sulfatide, other self-glycolipids, β-GlcCer, β-GalCer, and lysophosphatidylcholine (LPC) have been reported to be recognized by type II NKT cells (10, 23, 24) (Figure 1). The lipid Ags from microbial sources, such as glycolipids from *Mycobacterium tuberculosis* or *Corynebacterium glutamicum* (11) and phosphatidylglycerol from *Listeria monocytogenes* (25), have also been reported to be Ags for type II NKT cells other than those described above. Observations that some Ags recognized by a fraction of type II NKT cells or type II NKT cell hybridomas are not always recognized by other fractions of the cells suggest that the type II NKT cell population contains multiple cell subsets specific for distinct Ags. However, the functional diversity of type II NKT cells recognizing distinct Ags remains to be explored.

## Type II NKT Cells in Humans

Human type II NKT cells have been studied as CD1d-reactive T cells expressing diverse TCRs and were found to be more frequent than type I NKT cells in bone marrow, liver, and inflamed intestines of patients with ulcerative colitis (26–28).

Also, some lipid ligands have been reported to be recognized by both murine and human type II NKT cells, such as β-GlcCer 22:0, glucosylsphingosine (29), sulfatide, and lysosulfatide (30, 31). LPC was discovered from the plasma of myeloma patients and has been shown to be recognized by both human and murine type II NKT cells (24, 32–34). Human type II NKT cells are also reported to recognize non-lipid small molecules, such as phenyl pentamethyldihydrobenzofuran (35) (Figure 1). Interestingly, although most lipid ligands for type II NKT cells are not recognized by type I NKT cells, LPC is reported to be recognized by a few human type I NKT cell clones. However, it is not recognized by murine type I NKT cells (32, 36–38).

In addition to the use of αβ-TCRs, NKT cells using γδ-TCRs have been described. In humans, γδ-T cells that recognize lipid Ag presented by CD1d were discovered in peripheral blood and nasal mucosa of cypress pollen-sensitive subjects (39–41). These cells recognize phospholipids, such as phosphatidylcholine and phosphatidylethanol, extracted from pollen grains (41) (Figure 1). Surprisingly, although sulfatide-reactive type II NKT cells in mice use αβ-TCRs, at least some sulfatide-reactive CD1d-restricted T cells in humans have been shown to express γδ-TCRs (42, 43). In addition, although sulfatide has been considered as a specific Ag for type II NKT cells, a recent report demonstrated that human, but not mouse, type I NKT cells could recognize sulfatide presented by CD1d (44). These findings suggest that sulfatide-reactive type II NKT cells in humans and mice may comprise distinct populations, so we may need to subdivide them for further analysis.

## The Mechanism of Type II NKT Cell-Mediated Suppression of Tumor Immunity

The initial observation that NKT cells can suppress tumor immunity was reported in 2000 (15). In a 15-12RM fibrosarcoma tumor model in which tumors show a growth-regression-recurrence pattern, IL-13 had a key role for downregulation of CTLs, and CD1d^−/−^ mice had decreased IL-13 production and resistance to the recurrence. Subsequently, type II NKT cells were shown to be sufficient for the suppression of tumor immunity in multiple mouse tumor models, in which CD4^+^CD25^+^ regulatory T cells (Tregs) do not play a critical role in the regulation of immunosurveillance (24, 45).

These represented indirect evidence of suppressive roles of type II NKT cells, which were obtained by comparing WT mice, Jα18^−/−^ mice, and CD1d KO^−/−^ mice. After the discovery of sulfatide as a ligand for type II NKT cells, direct evidence of the suppressive role of type II NKT cells was obtained. The administration of sulfatide to activate sulfatide-reactive type II NKT cells enhanced tumor growth in a CD1d-dependent manner in a murine colon cancer cell line, CT26, lung metastasis model (46). Although it is not clear whether all type II NKT cells suppress tumor immunity, the studies suggest that type II NKT cells in the absence of type I NKT cells in Ja18^−/−^ mice are sufficient to suppress tumor immunity. However, among subsets of type II NKT cells, we have detailed knowledge only of sulfatide-reactive ones, and even among those, we cannot be sure that all of them are immunosuppressive.

**Figure 2:**
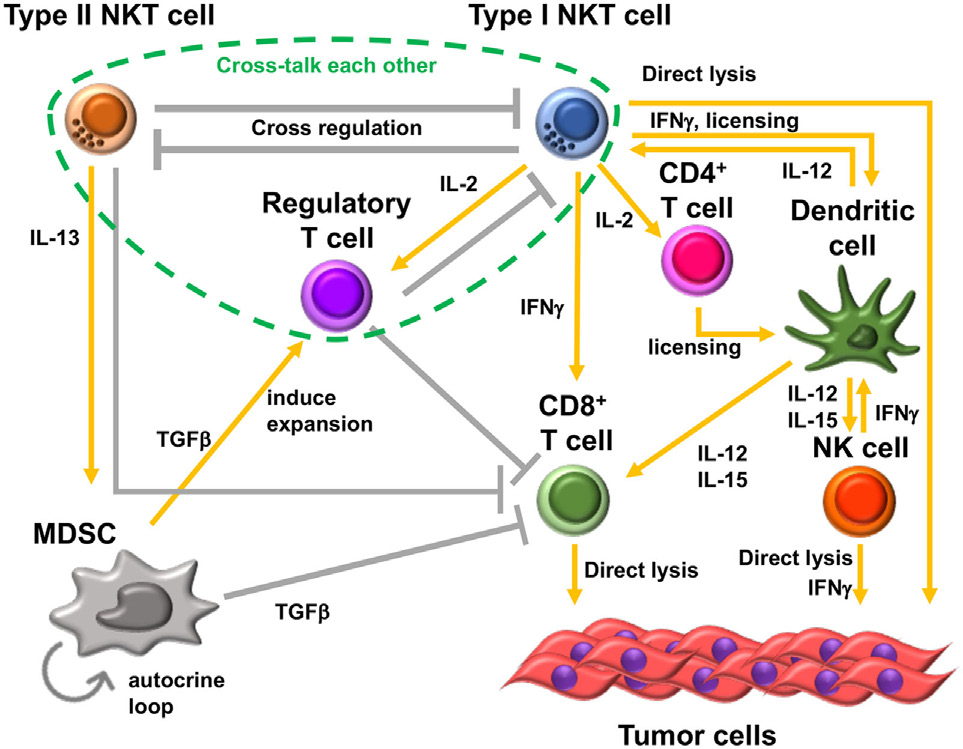
Immunosuppressive cell network in tumor microenvironment. Type II natural killer T (NKT) cells and type I NKT cells cross-regulate each other. Type II NKT cells cross talk with myeloid-derived suppressor cells (MDSCs) through production of IL-13. Transforming growth factor beta (TGF-β) produced by MDSCs suppresses CD8^+^ T cells, induces immunosuppressive regulatory T cells (Tregs), and enhances development of additional tumor-associated MDSCs by an autocrine loop. In addition, type II NKT cells can suppress CD8^+^ T cells by unknown mechanisms other than through cross talk with MDSCs. Type I NKT cells enhance the antitumor function of CD8^+^ T cells and are able to directly lyse tumor cells. Type I NKT cells’ interaction with dendritic cells (DCs) through CD1d-TCR and CD40-CD40L induces activation and maturation of DCs. The interaction licenses DCs to be able to prime CD8^+^ T cells and produce IL-12 and IL-15. IL-12 and IL-15 production by DCs stimulates natural killer (NK), type I NKT, and/or CD8^+^ T cells. IL-2 produced by activated type I NKT cells induces the proliferation of memory CD4^+^ T cells, which support the activation of CD8^+^ T cells. However, type I NKT cells also support Tregs through IL-2 production. Treg cells suppress type I NKT cells and CD8^+^ T cells. Three kinds of T cells, type II NKT cells, type I NKT cells, and Tregs, cross talk with each other.

One of the mechanisms of type II NKT cell-mediated suppression is through cross talk with myeloid-derived suppressor cells (MDSCs). In the 15-12RM fibrosarcoma tumor model, although IL-13 was necessary for downregulation of CTL-mediated tumor immunosurveillance, it could not directly downregulate CTL activity as T cells do not have receptors for IL-13. IL-13 induced transforming growth factor beta (TGF-β) production by the CD11b^+^Gr1^+^ population of myeloid suppressor cells, and blocking TGF-β or depleting Gr1^+^ cells *in vivo* could inhibit the suppression of tumor immunity by type II NKT cells (47) (Figure 2). This IL-13 signaling is mediated through an IL-4 receptor α, which forms a heterodimer with IL-13 receptor α1, and the STAT6 pathway. There is another receptor that can bind IL-13, IL-13 receptor α2, whose expression is induced by tumor necrosis factor-α (TNF-α) along with the STAT6 signal from IL-4 or IL-13. Because a TNF-α-neutralizing agent was shown to be able to inhibit the suppression of tumor immunity by type II NKT cells, TNF-α is also involved in this signaling pathway (48). It was shown that induction of TGF-β requires a two-step process in which TNF-α and IL-13/4/STAT6 synergistically upregulated the IL-13Rα2, which then responded to IL-13 to induce TGF-β production through AP-1 signaling. This interaction with MDSCs was also reported in a CD1d-overexpressing B cell lymphoma model (49). Interestingly, reports of similar IL-13-mediated cross talk with MDSCs by group 2 innate lymphoid cells have been recently published (50, 51). Therefore, this IL-13-mediated cross talk with MDSCs is not limited to type II NKT cells, and multiple kinds of immune cells that can produce IL-13 may be involved in this immunosuppressive loop. However, it should be pointed out that CD1^−/−^ mice that lack NKT cells with facilitated tumor immunity retain ILCs, so the effects lost in these mice must be dependent on NKT cells rather than ILCs.

Another mechanism may exist for suppression of tumor immunity by type II NKT cells. In a K7M2 mouse osteosarcoma model, CD1d^−/−^ mice showed higher resistance to growth of osteosarcoma primary tumors than WT mice. The protection was shown to be CD8^+^ T cell dependent, and CD1d^−/−^ mice had significantly higher numbers of tumor-infiltrating lymphocytes. In this model, TGF-β and IL-13 were not the drivers of immunosuppression (52). Thus, alternative pathways exist for immunosuppression mediated by type II NKT cells.

## Cross-Regulation of Type I NKT Cells and Type II NKT Cells

As mentioned above, type I and type II NKT cells generally have opposite function in tumor immunity. In addition, these two subsets of NKT cells cross-regulate each other (Figure 2). In the CT26 pulmonary metastasis model, selective stimulation of type II NKT cells by sulfatide enhanced tumor growth. In addition, when both type I and type II NKT cells were activated simultaneously by α-GalCer and sulfatide, respectively, the tumor immunity by activated type I NKT cells was inhibited by concurrent activation of type II NKT cells. This finding suggested that activated type II NKT cells may suppress type I NKT cell-mediated enhancement of tumor immunity (46). Analogously, in *in vitro* analysis, α-GalCer-induced cytokine production by type I NKT cells was inhibited by type II NKT cells stimulated with sulfatide (46, 53). This suppressive effect of type II NKT cells against type I NKT cells was also reported in mouse models of other diseases. For example, in a model of concanavalin A-induced hepatitis, activation of type II NKT cells by sulfatide induced anergy, or hyporesponsiveness, of type I NKT cells (54).

In the CT26 pulmonary metastasis model, Jα18^−/−^ mice showed lower and CD1d^−/−^ mice showed higher resistance to tumor growth than WT mice. Consistent with this finding, Jα18^−/−^ mice showed a weaker specific CTL response than did WT mice against tumor Ag-pulsed cells, whereas CD1d^−/−^ mice showed a stronger tumor-Ag-specific cytotoxic response than WT mice. These results suggested that type I NKT cells inhibit the function of type II NKT cells in tumor immunity to suppress CTL activity (46). Altogether, therefore, type I and type II NKT cells form a novel immunoregulatory axis of cells with opposing roles that counteract each other, in which the balance affects the tone of local immunity, in that sense (but not in specific cytokines) analogs to the Th1–Th2 axis that so profoundly affected immunology (55). Because NKT cells act early in immune responses, the balance along this axis can have profound effects on subsequent conventional T cell or antibody responses (56).

## Cross Talk among Three Types of Immunoregulatory T Cells

In addition to cross-regulation of two subsets of NKT cells, type II NKT cells may also cross talk with CD4^+^Foxp3^+^ Tregs. In a CT26 subcutaneous tumor model, this cross-regulation of two subsets of NKT cells helps determine the primary suppressive cell in tumor immunity. Although Tregs are known not to have suppressive effects in the CT26 pulmonary metastasis model, they do have a suppressive effect in the CT26 subcutaneous tumor model. In the CT26 subcutaneous tumor model, Treg blockade was sufficient to protect against tumor outgrowth in WT type and CD1d^−/−^ mice. However, Treg blockade was insufficient for protection in Jα18^−/−^ mice in which type II NKT cells are unopposed (57, 58). It was demonstrated that in WT mice, type II NKT cells are neutralized by type I NKT cells, leaving Tregs as the primary suppressor in this model. In mice lacking type I NKT cells, in which type II are not neutralized by type I NKT cells, type II NKT cells could suppress tumor immunity even when Treg cells are blocked. Thus, type I NKT cells regulate the balance between other regulatory cells, regulating the regulators. This situation may apply in human cancer patients, as it was reported that myeloma patients have deficient type I NKT cell function (59), and other studies of type I NKT cells in cancer patients have reported either decreased numbers or decreased cytokine production (59–64). This altered balance of type I versus II NKT cells in patients with cancer may affect the dominant immune-suppressive cells in the patients.

## Potential Role of Type II NKT Cell in Chronic Inflammation-Mediated Cancer

Type II NKT cells may play an important role in inflammation-induced cancers. Because type II NKT cells react to endogenous lipids, they may be activated when endogenous lipids are released from autologous organs damaged by inflammation. In addition, unusual lipid accumulation caused by metabolic disorders may induce the activation of type II NKT cells. Gaucher’s disease (GD) is an inherited metabolic disorder characterized by lysosomal storage of β-GlcCer (d18:1/C22:0; βGL1-22) and glucosylsphingosine (Lyso-GL1; LGL1) (65). It has been reported that the overall cancer risk is increased in GD patients (66–68). Especially, the association of GD and multiple myeloma is most striking, with the risk estimated at almost 37-fold compared to the general population (68).

Studies in human GD patients revealed that human type II NKT cells react to βGL1-22 and LGL1 that accumulate in these patients. Also, both βGL1-22/LGL1-reactive type II NKT cells express markers of T follicular helper cells (CXCR5^hi^ PD1^hi^ ICOS^hi^ BCL6^+^IL-21^+^) and promote plasma cell differentiation in human T-B cocultures (29). In addition, it is reported that the clonal immunoglobulin in patients with GD is reactive against LGL1. Furthermore, administration of anti-LGL1 antibodies ameliorates GD-associated gammopathy in mice, suggesting that long-term immune activation by LGL1 may underlie GD-associated gammopathies (69). These findings suggest that type II NKT cells are activated due to abnormal accumulation of lipid Ags and provide help for B cell activation in patients with GD. This chronic lipid-mediated and type II NKT cell-mediated B cell activation may underlie the increased risk of plasma cell tumors in GD.

Notably, dysregulation of glucosphingolipids has been demonstrated not only in inherited metabolic disorders but also in obesity (70). Recently, obesity is viewed as a chronic low-grade inflammatory disease that is also associated with cancer risk (71). The relationship between NKT cells and obesity is unclear, because three different outcomes for the involvement of NKT cells in the development of obesity are reported. Some groups reported a protective role and demonstrated that type I NKT cells in adipose tissue produce anti-inflammatory cytokines, such as IL-4 and IL-10 (72–74). On the other hand, other groups reported their aggravating role and demonstrated that type I NKT cells produced pro-inflammatory cytokines, such as IFN-γ, in response to lipid excess in the body (75, 76). In addition, another group reported a neutral role and stated NKT cells have no active role for skewing the environment toward either a Th1- or Th2-bias during the development of obesity (77). Regarding the involvement of type II NKT cells, one of the reports demonstrated that type II NKT cells exacerbated diet-induced obesity in the absence of type I NKT cells (78). Thus, type II NKT cells may be activated during obesity-induced chronic inflammation and may have a role for exacerbation of obesity and carcinogenesis associated with obesity.

## Potential Role of Type II NKT Cells in Cancer Immunoediting

In the tumor microenvironment, cancer cells affect and modulate antitumor immunity to escape immunosurveillance. Some cancer cells are reported to express CD1d, suggesting that they may affect NKT cell-mediated antitumor immunity. Considering that type I NKT cells have been reported to be able to eliminate CD1d-expressing tumor cells *via* multiple pathways (79–83), it seems that CD1d on the cancer cells is mainly recognized by type I NKT cells, resulting in enhancement of tumor immunity. However, conversely, CD1d expression in human cancer has been reported to be correlated with poor prognosis in human renal cell carcinoma and multiple human hematopoietic malignancies (84–87). This finding suggests that CD1d on the cancer cells may be recognized by type II NKT cells and that activated type II NKT cells may induce suppression of tumor immunity.

## TGF-β Blockade as a Therapeutic Application of Blocking Type II NKT Cell-Mediated Immune Suppression

One of the candidates for therapeutic targets for cancer immunotherapy is type II NKT cell-MDSC cross talk, which results in TGF-β-mediated CTL downregulation. A role for TGF-β in cancer-mediated immunosuppression was demonstrated in 1990 for the first time (88). Initial studies to inhibit TGF-β signaling by antibodies demonstrated enhanced cancer cell-specific immune responses (89) and reduced tumorigenicity of a human breast cancer cell line in athymic mice (90).

In addition, to enhance the effect of TGF-β blockade on tumor immunity, combination therapies have been studied. In a murine B16 melanoma model, neutralizing antibodies to TGF-β combined with IL-2 therapy could decrease the number of metastases (91). Similar synergistic effect of TGF-β blockade in conjunction with a cancer vaccine has been reported in multiple tumor models with multiple vaccine platforms (92–97). In humans, an antibody that neutralizes all three isoforms of active TGF-β has shown clinical benefit in some patients with metastatic malignant melanoma (98, 99). Of course, TGF-β can be made by many cell types, not just MDSCs. For example, TGF-β can be important in induction of some types of Treg cells and can play a role in their function (100). It can also be made by tumor cells themselves. Thus, TGF-β blockade can promote antitumor immunity through a plethora of complementary mechanisms. Nevertheless, despite their limited numbers, type II NKT cells may play a key role, as a frequency of as much as 4.5% of sulfatide-reactive type II NKT cells in the liver (7) is actually a substantial number when one considers that the steady-state frequency of conventional T cells with a single-Ag specificity is orders of magnitude lower.

In addition to synergy with IL-2 therapy or a cancer vaccine, TGF-β blockade may have multiple benefits for the induction of adequate immune response to tumor cells. Because TGF-β is a pleiotropic cytokine, it has multiple roles in tumor immunity. First, as mentioned above, TGF-β production by MDSCs directly suppresses other immune cells, such as CTLs. Second, TGF-β produced by MDSCs also feeds into an autocrine loop to enhance the development of additional tumor-associated MDSCs (101). Third, MDSCs induce expansion of immunosuppressive, tumor-specific Tregs (102), resulting in stronger suppression of CTLs. Altogether, TGF-β blockade could interrupt these autocrine and paracrine loops driving suppression of tumor immunity (101, 103).

## Other Possible Therapeutic Applications of Blocking Type II NKT Cell-Mediated Immune Suppression

One of the possible therapeutic approaches targeting type II NKT cell-mediated immune suppression is development of an antagonistic Ag for type II NKT cells. The development of antagonistic Ags for type II NKT cells that have higher affinity for CD1d than tumor Ags may enable blocking the signaling between tumor lipid Ags and type II NKT cells. The candidates for the antagonistic Ags are structural analogs of type II NKT cell Ags since it is reported that the affinity between lipid Ag and CD1d differ according to the structure of the fatty acid chain of the glycolipid Ags (104, 105). To this end, it is important to carry out structure–function studies of the tumor-derived and other lipid Ags recognized by type II NKT cells in cancer patients. Such studies are underway in our laboratory.

## Conclusion

After development of immunotherapy targeting a CTLA-4 or a PD-1 signaling pathway, multiple combination therapies have been studied. More detailed understanding of the roles and cross talk among immune cells in the tumor microenvironment will be necessary for the development of effective combination therapies. In addition to the more widely studied immunoregulatory cells such as Tregs and MDSCs, here we have reviewed abundant evidence that type II NKT cells play a major role in regulating immunity against cancer. Furthermore, the dominant immunosuppressive cells may differ among different types of cancer or sites of metastasis. Thus, the development of diagnostic methods to determine dominant immunosuppressive cells and proper targeting of cells or pathways for individual patients is needed to relieve this suppression and allow the full efficacy of the immune system to be marshaled to treat cancer. This “precision diagnosis of immunosuppressor cells in the tumor microenvironment” would help enhance the efficacy and decrease adverse effects of cancer immunotherapy.

## Author Contributions

All authors listed have made a substantial, direct and intellectual contribution to the work and approved it for publication.

## Conflict of Interest Statement

The authors declare that the research was conducted in the absence of any commercial or financial relationships that could be construed as a potential conflict of interest.
